# Comparative Longitudinal Serological Study of Anti-SARS-CoV-2 Antibody Profiles in People with COVID-19

**DOI:** 10.3390/microorganisms11081985

**Published:** 2023-08-02

**Authors:** Marilou H. Barrios, Suellen Nicholson, Rowena A. Bull, Marianne Martinello, William Rawlinson, Michael Mina, Jeffrey J. Post, Bernard Hudson, Nicole Gilroy, Andrew R. Lloyd, Pamela Konecny, Francesca Mordant, Mike Catton, Kanta Subbarao, Leon Caly, Julian Druce, Hans J. Netter

**Affiliations:** 1 Victorian Infectious Diseases Reference Laboratory (VIDRL), The Royal Melbourne Hospital, Melbourne, VIC 3000, Australia; mehercia@gmail.com (M.H.B.); suellen.nicholson@vidrl.org.au (S.N.); cattonms2@gmail.com (M.C.); leon.caly@mh.org.au (L.C.); julian.druce@mh.org.au (J.D.); 2 Peter Doherty Institute, University of Melbourne, Melbourne, VIC 3000, Australia; francesca.mordant@unimelb.edu.au (F.M.); kanta.subbarao@influenzacentre.org (K.S.); 3 The Kirby Institute, University of New South Wales (UNSW), Sydney, NSW 2052, Australia; r.bull@unsw.edu.au (R.A.B.); mmartinello@kirby.unsw.edu.au (M.M.); a.lloyd@unsw.edu.au (A.R.L.); 4 School of Biomedical Sciences, Faculty of Medicine and Health, University of New South Wales (UNSW), Sydney, NSW 2052, Australia; w.rawlinson@unsw.edu.au; 5 Serology and Virology Division, Department of Microbiology, New South Wales Health Pathology, Randwick, Sydney, NSW 2031, Australia; 6 Prince of Wales Hospital, Sydney, NSW 2031, Australia; jeffrey.post@health.nsw.gov.au; 7 Northern Beaches Hospital, Frenchs Forest, NSW 2086, Australia; michaelmina2001@yahoo.com; 8 School of Clinical Medicine, University of New South Wales (UNSW), Sydney, NSW 2052, Australia; pam.konecny@health.nsw.gov.au; 9 Royal North Shore Hospital, Sydney, NSW 2065, Australia; bernard.hudson@health.nsw.gov.au; 10 Westmead Hospital, Sydney, NSW 2145, Australia; nicky.gilroy@health.nsw.gov.au; 11 St. George Hospital, Sydney, NSW 2217, Australia; 12 Department of Microbiology and Immunology, University of Melbourne, Melbourne, VIC 3000, Australia; 13 World Health Organization Collaborating Centre for Reference and Research on Influenza at the Peter Doherty Institute, Melbourne, VIC 3000, Australia; 14 School of Science, Royal Melbourne Institute of Technology (RMIT) University, Melbourne, VIC 3001, Australia

**Keywords:** SARS-CoV-2, serology, antibody profile, polyclonal durability

## Abstract

Serological diagnostic assays are essential tools for determining an individual’s protection against viruses like SARS-CoV-2, tracking the spread of the virus in the community, and evaluating population immunity. To assess the diversity and quality of the anti-SARS-CoV-2 antibody response, we have compared the antibody profiles of people with mild, moderate, and severe COVID-19 using a dot blot assay. The test targeted the four major structural proteins of SARS-CoV-2, namely the nucleocapsid (N), spike (S) protein domains S1 and S2, and receptor-binding domain (RBD). Serum samples were collected from 63 participants at various time points for up to 300 days after disease onset. The dot blot assay revealed patient-specific differences in the anti-SARS-CoV-2 antibody profiles. Out of the 63 participants with confirmed SARS-CoV-2 infections and clinical COVID-19, 35/63 participants exhibited diverse and robust responses against the tested antigens, while 14/63 participants displayed either limited responses to a subset of antigens or no detectable antibody response to any of the antigens. Anti-N-specific antibody levels decreased within 300 days after disease onset, whereas anti-S-specific antibodies persisted. The dynamics of the antibody response did not change during the test period, indicating stable antibody profiles. Among the participants, 28/63 patients with restricted anti-S antibody profiles or undetectable anti-S antibody levels in the dot blot assay also exhibited weak neutralization activity, as measured by a surrogate virus neutralization test (sVNT) and a microneutralization test. These results indicate that in some cases, natural infections do not lead to the production of neutralizing antibodies. Furthermore, the study revealed significant serological variability among patients, regardless of the severity of their COVID-19 illness. These differences need to be carefully considered when evaluating the protective antibody status of individuals who have experienced primary SARS-CoV-2 infections.

## 1. Introduction

Coronavirus disease (COVID-19) is caused by the severe acute respiratory syndrome coronavirus 2 (SARS-CoV-2) and exhibits a range of symptoms that can vary from person to person. The manifestations range from asymptomatic to mild or severe illness and can include respiratory illness, muscle and joint pain, abdominal pain, and diarrhea [1,2]. Approximately one-third of SARS-CoV-2 infections are asymptomatic [2,3] and most people with symptoms develop mild-to-moderate illnesses (81%). Severe illness (14%) presentations include dyspnoea, hypoxia with over 50% lung involvement, and critical illness that occurs in 5% of cases and manifests as respiratory failure and multiorgan system dysfunction [4]. The SARS-CoV-2 virions consist of four structural proteins: the membrane (M), envelope (E), nucleocapsid (N), and spike (S) proteins [5]. Among the coronavirus structural proteins, the S and N proteins are significant targets for the immune system and hence are important components of serological assays [6]. The S protein is a glycosylated type I membrane protein, which is anchored and protrudes from the virus envelope. S proteins form homotrimers and facilitate virus entry into the host cell using the angiotensin-converting enzyme 2 (ACE2) receptor [7,8]. The S precursor protein is cleaved into the S1 and S2 domains via a furin-like protease. The S1 domain contains the exposed receptor-binding domain (RBD), which engages the ACE2 receptor. The S2 domain mediates the fusion of viral and host membranes [9]. The S protein is highly immunogenic with RBD as a target of neutralizing antibodies [7].

The global circulation of SARS-CoV-2 and its zoonotic potential strongly suggest that the elimination of the virus is not achievable [10,11]. Active case management and current case definition for SARS-CoV-2 infection are based on nucleic acid tests (real-time reverse transcription polymerase chain reaction, RT-PCR) or rapid antigen-detecting tests (RATs) using upper or lower respiratory tract samples, but sensitivity depends on viral load, sampling location, and technique [12,13,14]. Unlike the detection of viral RNA by RT-PCR or viral proteins by RAT, antibodies targeting SARS-CoV-2 persist beyond the acute infection phase. The serological diagnostic assays to measure antibody responses play an essential role in epidemiological surveillance, quantifying the prevalence of infections in a community, assessing immunity in a population as an outbreak progresses, and monitoring the effectiveness of vaccination [15]. Different serological assays have been developed, such as enzyme-linked immunosorbent assays (ELISAs), electrochemiluminescence immunoassays, surrogate neutralization assays, and assays based on dot blot techniques [16,17,18,19,20,21]. The combined use of different viral antigens in a multiplex dot blot assay allows the assessment of the serological status of patients with high sensitivity [18]. In a subset of patients, anti-SARS-CoV-2 antibodies were detected within one week of disease onset, and the number of patients who seroconverted substantially increased 14 days or later after the onset of symptoms [22,23]. SARS-CoV-2 infection triggers a rapid antibody response in people with or without symptoms [24,25]. A population-based study conducted in Spain demonstrated that at least one-third of individuals with anti-SARS-CoV-2 antibodies were asymptomatic [26]. The epidemiological and serological evaluations underscore the significance of gaining an enhanced understanding of the connection between antibody response and disease severity. This understanding is crucial for establishing a strong prognostic relationship.

The use of antigen combinations or a panel of antigen-specific assays is essential to minimize false-positive rates, differentiate between various persistent antigen-specific antibodies, including those with neutralizing capabilities, and ultimately establish comprehensive antibody profiles. Anti-RBD and anti-N protein antibodies were detected within four days after symptom onset, followed by the detection of anti-S1 and anti-S2 antibodies around 13 days post-symptom onset [27]. Antibodies targeting RBD and S1 or S2 seem to persist longer than anti-N protein antibodies, which often become undetectable within five to seven months [28]. On the other hand, studies conducted over an eight-month period have shown the detection of antibodies targeting the S and RBD proteins [29,30]. Optimal antigen combinations for serological analyses have been determined, which include S1 and RBD for both IgG and IgM antibodies, S2 for IgM, and N-protein for IgG [31]. Although elevated antibody titres to S1, RBD and N proteins have been associated with severe diseases and worse clinical classifications [22,23,28], the presence of antibodies targeting RBD has been shown to strongly correlate with virus-neutralizing activity by interfering with the binding of RBD to the ACE2 receptor [30,32,33]. Taken together, these studies have demonstrated the significance of multiplexing serological assays and the importance of longitudinal studies for determining the quality of the resulting immune response.

To obtain a comprehensive view of the diversity and quality of the anti-SARS-CoV-2 antibody response specific for patients with different disease severities, we developed a dot blot immunoassay that includes different individual S protein domains, S1, RBD, S2, and the N protein. This assay enabled us to measure the patients’ antibody signatures in a pre-vaccination cohort. Anti-SARS-CoV-2 sera were taken at different time points after the disease onset, allowing the assessment of the polyclonal durability of the response and whether antibody profiles correlate with disease severity. The immune reactivity of the sera against the selected antigen targets revealed patient-to-patient differences in the antibody profiles and identified individuals with low or undetectable levels of anti-S1, RBD, and S2 antibodies. This study highlights the importance of employing multiple antigen targets for screening convalescent serum samples, allowing the identification of individuals with past SARS-CoV-2 infections and potentially reduced humoral immune protection.

## 2. Materials and Methods

### 2.1. Plasmids

The SARS-CoV-2 cDNA sequences were based on the genomic sequence of the reference isolate Wuhan-Hu-1 (accession MN908947, National Center for Biotechnology Information (NCBI), Bethesda, MD, USA). Sequences of genes encoding S1, S2, RBD, and N were synthesized using GenScript (Piscataway, NJ, USA) and cloned into a pCAGGS vector for expression in mammalian cell lines. The plasmid pCW-5 encoded the S1 domain of the spike protein from amino acid (aa) position 1 to position 672, pCW-16 encoded the S2 domain (aa 673–1198 in the absence of the transmembrane region and internal sequence), and pCW18 encoded the RBD (amino acid 307–527). The mouse interleukin 3 signal sequence was expressed upstream of the open reading frames for the S1, RBD, and S2 domains to facilitate secretion into the cell culture medium. The S1, RBD, and S2 domains, including the N protein (pCW-3), contained a FLAG-tag that was used to purify the proteins from the cell culture medium (S1, RBD, and S2 protein domains) or cell lysate (N protein).

### 2.2. Cell Culture

The human embryonic kidney (HEK293T) cell line was maintained in Dulbecco’s minimal essential medium (DMEM) and used for the expression of the S1, RBD, S2, and N proteins. The DMEM medium was supplemented with 10% fetal bovine serum (FBS) (Gibco, ThermoFisher Scientific, Waltham, MA, USA), penicillin, and streptomycin (100 μg/mL). Cells were maintained in 5% CO_2_ at 37 °C.

### 2.3. Production of SARS-CoV-2 Proteins

The HEK293T cells in 100 mm dishes were transfected at 70–80% confluency with 8 μg plasmids expressing SARS-CoV-2 specific gene products using Fugene 6 reagent (Promega, Madison, WI, USA) and Opti-MEM reduced serum medium (Gibco, ThermoFisher Scientific, Waltham, MA, USA). Supernatants were collected on days 3 and 6 and centrifuged at 300× *g* at 4 °C to remove cellular debris. Supernatants were purified via affinity chromatography. Meanwhile, cell lysates were collected on day 6 by initially washing the cells with cold PBS and then lysed with chilled lysis buffer (50 mM Tris-HCl, pH 7.5, 150 mM NaCl, 5 mM NaCl, and 1% NP-40 supplemented with protease inhibitor cocktail). Lysed cells were spun at 16,200× *g* for 15 min at 4 °C, and the supernatant was collected and stored at −20 °C until use.

### 2.4. Purification of SARS-CoV-2 Proteins

The SARS-CoV-2 proteins (RBD, S1, and S2) were purified from the cell culture medium, and the N protein was purified from the cell lysate using the FLAG M2 purification kit (CELLMM2, Sigma-Aldrich, St. Louis, MO, USA). Briefly, 10 mL of supernatant or 1 mL lysate (approximately 10 million cells) was combined with 1 mL anti-FLAG M2 resin (Sigma-Aldrich, St. Louis, MO, USA) for batch purification and incubated on a rotating wheel for 1.5 h at room temperature. The beads solution was then poured into a chromatography column for washing and eluting. Unbound proteins were eluted by washing with 20 mL of wash buffer. Consequently, bound proteins were eluted by FLAG competition using FLAG peptides (100–250 ng) dissolved in 500 μL of 1× wash buffer. Each FLAG peptide was incubated in the column for 30 min to elute bound proteins. Excess FLAG-peptide was removed using an Amicon Ultra 10 kDa filter (Merck Millipore, Darmstadt, Germany).

### 2.5. Dot Blot

RBD (500 ng), S1 (250 ng), S2 (100 ng), and N (100 ng) in 20 μL of Tris buffer (0.05 M Tris HCl; pH 7.4; 0.15 M NaCl) were dotted onto a pre-wet supported nitrocellulose membrane using Bio-Dot apparatus (BioRad, Hercules, CA, USA). Dot blot strips were cut and individually placed into disposable polypropylene trays, blocked with 5% non-fat dry milk in Tris/HCl buffer (pH 7.4) for 1 h, washed 3 times for 10 min with Tris/HCl buffer, and then probed with 20 μL of heat-inactivated COVID-19 patient sera in 2 mL of Tris/HCl buffer for 2 h. After another wash for 30 min, monoclonal anti-human IgG-HRP (Abcam, Cambridge, UK) was added and incubated for 1 h. Dot blots were visualized using an ECL reagent (Perkin Elmer, Waltham, MA, USA) and imaged using a ChemiDoc imaging system (BioRad, Hercules, CA, USA). Each dot was visualized by chemiluminescence, the intensity was measured using ImageJ software, and values were displayed as integrated density.

### 2.6. Commercially Available Immunoassays

The Wantai BioPharma (Beijing, China) ELISA was used for the detection of immunoglobulins IgM or total Ig of SARS-CoV-2 RBD, and Roche immunoassay was used to examine anti-S total Ig (Elecsys anti-SARS-CoV-2 S (RBD)) (Roche, Basel, Switzerland). The GenScript surrogate virus neutralization test (sVNT) (Piscataway, NJ, USA) was used to detect the presence of antibodies interfering with RBD binding to the ACE2 receptor. For the GenScript sVNT assay, 30 to <60% was regarded as low, 60% to 90% as medium, and >90% as high levels of potentially neutralizing antibodies. The tests were performed according to the manufacturer’s instructions (https://www.genscript.com/covid-19-detection-svnt.html) (accessed on 26 May 2023) and as described by Tan et al. [34].

### 2.7. Microneutralization Assay

SARS-CoV-2 isolate CoV/Australia/VIC01/202042 was passaged in Vero cells and stored at −80 °C. Plasma was heat-inactivated at 56 °C for 30 min. Plasma was serially diluted 1:20 to 1:10240 before the addition of 100 TCID50 of SARS-CoV-2 to MEM/0.5% BSA and incubation at room temperature for 1 h. Residual virus infectivity in the plasma/virus mixtures was assessed in quadruplicate wells of Vero cells incubated in serum-free media containing 1 µg/mL TPCK trypsin at 37 °C/5% CO_2_; the viral cytopathic effect was measured on day 5. The neutralizing antibody titre was calculated using the Reed/Muench method [35,36]. All the samples were assessed using two independent microneutralization assays.

### 2.8. Participant Samples

Participant samples were collected from the COSiN (Collection of COVID-19 Outbreak Samples in New South Wales, Australia), The Kirby Institute, NSW, Australia. The COSiN study is an ongoing prospective cohort evaluating the natural history of SARS-CoV-2 infection among adults and children in New South Wales, Australia [37]. People diagnosed with SARS-CoV-2 infection were eligible for enrolment, irrespective of disease severity. Participants were enrolled from seven hospital in- and outpatient departments and referred to microbiology laboratories in New South Wales between 6 March 2020 and 17 September 2020 before the COVID-19 vaccine rollout in Australia in February 2021. Eighty-one participants were included in this study, and sera from 63 patients were accessible, with a median age of 57 years (IQR: 71–40 years) (33 female/30 male) (Supplementary Table S1). Sera from participants were taken at different time points during 0–100, 101–200, and 201–300 days, following symptom onset or date of diagnosis (whichever was first). At each visit, clinical data and blood samples were collected. Disease severity was classified according to the NIH stratification (https://www.covid19treatmentguidelines.nih.gov, accessed on 26 May 2023; section Overview, sub-section Clinical Spectrum) [37]. The clinical manifestations included mild illness: variety of symptoms such as fever, cough, sore throat, headache muscle pain nausea, and diarrhea but no shortness of breath or abnormal imaging; moderate illness: evidence of lower respiratory diseases with oxygen saturation measured using pulse oximetry SpO_2_ ≥ 94% in room air at sea level; severe and critical illness: SpO_2_ < 94% in room air at sea level, a respiratory rate > 30 breaths/min (severe), acute respiratory distress syndrome, exaggerated inflammatory response, thrombotic disease, and exacerbation of underlying conditions (critical).

### 2.9. Ethics Statement

The protocol was approved by the Human Research Ethics Committees of the Northern Sydney Local Health District and the University of New South Wales, NSW, Australia (ethics number ETH00520), and was conducted according to the Declaration of Helsinki and International Conference on Harmonization Good Clinical Practice (ICH/GCP) guidelines and local regulatory requirements. The approval for the prepandemic specimen was obtained from the Melbourne Health Human Research Ethics Committee (RMH HREC QA2020052) [38]. Written informed consent was obtained from all participants before the study procedures [37,38].

## 3. Results

### 3.1. Characterization of Anti-SARS-CoV-2 Antibody Profiles

**Figure 1:**
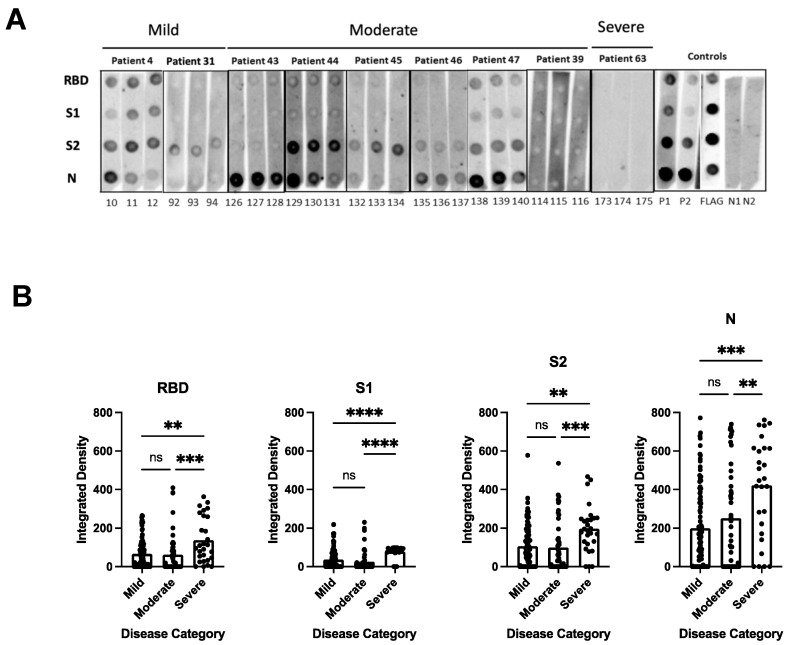
Dot blot analysis to assess patient antibody specificities for S1, RBD, S2 protein domains, and N protein. (**A**) Representative dot blots. Serum samples were derived from patients with mild (patients #4 and #31), moderate (patients #43, #44, #45, #46, #47 and #39), and severe (patient #63) COVID-19-related diseases. For sera from patients with mild and moderate disease, the first (strips 10, 92, 126, 129, 132, 135, 138 and 114), second (11, 93, 127, 130, 133, 136, 139, and 115), and third (12, 94, 128, 131, 134, 137, 140 and 116) serum samples were taken within the time periods of 0–100, 101–200, and 201–300 days, respectively. For patient #63 with severe disease, sera were taken on days 21, 23, and 29 days (strips 173, 174, and 175, respectively) after disease onset. Supplementary Table S1 shows a summary of all patient serum samples, with a total of 63 patients and 175 sera. Nitrocellulose-enhanced membranes were spotted with the individual proteins as indicated (**A**), incubated with 1:100 dilution of patient serum samples from mild, moderate, and severe COVID-19 patients, and then anti-human IgG-HRP secondary antibody was added. Dots were developed by chemiluminescence and images were taken. Integrated density was calculated by measuring the intensity of each dot minus the background. P1 = positive control 1 (1:100 dilution); P2 = positive control 2 (1:300 dilution); N = negative control; FLAG = strip probed with anti-FLAG antibody. (**B**) Antibody levels against individual RBD, S1, S2 protein domains, and N protein in relation to the different disease categories. The values plotted are the integrated density of each dot per antigen regardless of time points, age, and gender. The heights of the bar graphs represent the mean value of all individual measurements. Kruskal–Wallis test was conducted to compare the mean values of unpaired samples. *p* value: **** < 0.0001; *** < 0.0002; ** < 0.002; not significant (ns) < 0.12.

**Figure 2:**
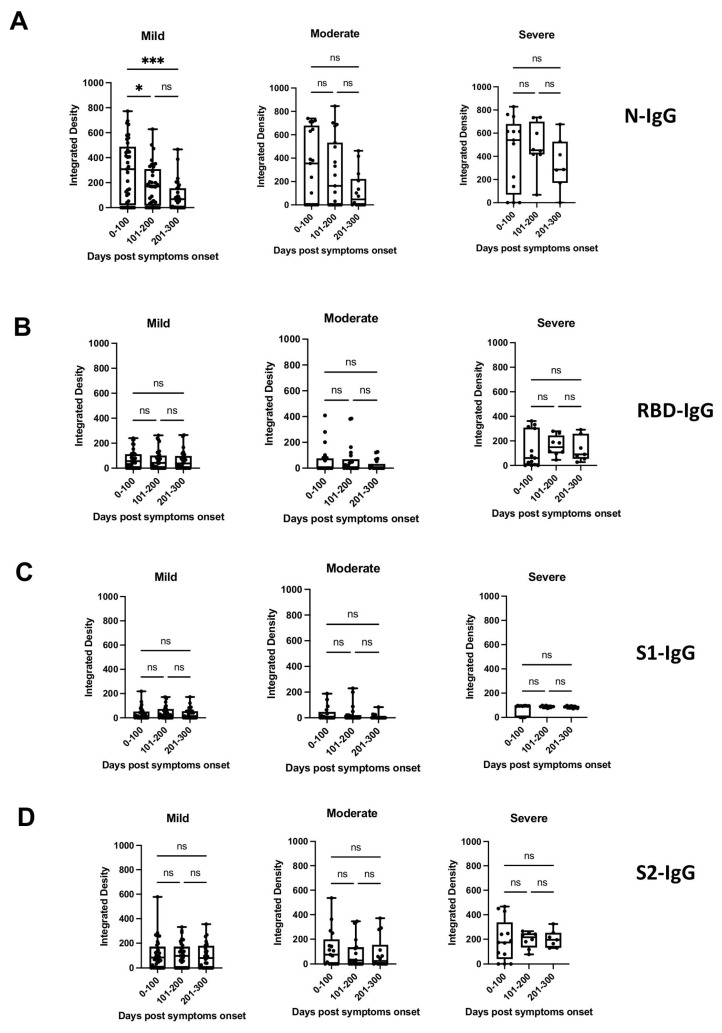
Presence of anti-N, anti-S1, anti-RBD, or anti-S2 antibodies (IgG) (panels (**A**–**D**), respectively) in patients with mild, moderate, or severe disease at different time periods post-symptom onset, as detected by dot blot analysis. Box plots were used to show the spread of the data. Data were analyzed using the analysis of variance (ANOVA) for paired samples. Mean values were compared using Dunn’s multiple comparisons test; *p* value: *** < 0.0002; * < 0.0332; not significant (ns) < 0.1234.

To determine the breadth of the anti-SARS-CoV-2 antibody response and to assess antibody persistence, we developed a dot blot assay to capture antibody profiles specific for different SARS-CoV-2 proteins and protein domains. The FLAG-tagged SARS-CoV-2 receptor-binding domain (RBD), spike S1 subdomain (S1), spike S2 subdomain (S2), and nucleocapsid (N) were probed using patient antiserum and tested for their IgG-specific reactivity for S1-, RBD-, S2-, and N proteins. Sixty-four pre-pandemic serum samples were used for the initial validation and were negative in the dot blot assay (data not shown). Longitudinal serum samples from 63 participants with COVID-19 were analyzed (median age 57 years [IQR 71–40 years]; female *n* = 33 [52%]; total serum samples *n* = 175) (Supplementary Table S1). One patient (#63) received Rituximab to treat unrelated COVID-19 conditions. The serum was taken from 32/63 participants with mild COVID-19 (total serum samples 97), 17/63 participants with moderate COVID-19 (total serum samples 50), and 14/63 participants with severe/severe-to-critical COVID-19 (total serum samples 28) [33], and arranged according to sample taking days post-symptom onset (DPSO, 0–100, 101–200, 201–300) (Supplementary Table S1). The integrated density measurements of the dot blots showed patient-specific antibody signatures against the S1, RBD, S2, and N protein antigens, revealing variations in the breadth of the anti-SARS-CoV-2 antibody response among different participants. The participants exhibited immunoreactivity against the complete set of antigens, some or none of the antigens. Most patients (35/63 patients) developed diverse and broad anti-S antibody responses (detection of S1, RBD, and S2 domains) in addition to the N protein (Figure 1A, patient sera #4, #47; Supplementary Table S1). However, 14/63 patients showed narrow antibody profiles with reactivities against only a subset of the provided target proteins, such as against the S2 domain and N protein (e.g., patient #45 and 46, Figure 1A and Supplementary Table S1), or exclusively against the N-protein (e.g., patient #25, Supplementary Table S1). Remarkably, two participants (#8 and #13) developed detectable levels of antibodies against RBD, S2, and N, but not against the S1 domain (Supplementary Table S1). This suggests that the generated anti-RBD antibodies possibly target an antigenic sequence that is not readily accessible within the S1 domain. Significantly higher levels of antibodies against the individual protein domains (S1, RBD, S2) and the N protein were observed in patients with more severe disease compared to patients with mild and moderate COVID-19 (*p* < 0.0001) (Figure 1B). The analyses of the sera taken at different time points after disease onset revealed that the antibody profiles persisted, and the antibody signatures were maintained during the test period. Participants with a broad immune response against the targets S1, RBD, S2, and N within the early time period (0–100 days after symptom onset) maintained the antibody signature at later time points (Figure 1A, Supplementary Table S1). Consistently, participants with restricted or immune-dominant responses maintained their specific signatures without any subsequent changes in their antibody profiles (Figure 1A, Supplementary Table S1). A declining trend in anti-N antibody levels was observed in the patient groups with moderate and severe diseases, but a significant decrease was observed in the group with mild disease (Figure 2). Patients positive for anti-S antibodies, including patients with low anti-S1, RBD, and S2 antibody levels, maintained their antibody levels for up to 300 days, irrespective of their disease severity (Figure 2B–D). Sera from 14/63 patients (approx. 20%) did not develop antibody levels detectable in the dot blot assay such as patients #39 and #63 (Figure 1A, Supplementary Table S1). Patient #63 received treatment with Rituximab, and hence lack of antibody formation was expected (Supplementary Table S2).

The dot blot analysis showed that regardless of the disease severity, patients developed broad or restricted (narrow) antibody profiles that persisted after symptom onset. The development of restricted antibody profiles or the absence of detectable levels of anti-S antibodies indicate that natural infections did not necessarily result in the formation of high levels of potentially neutralizing antibodies.

### 3.2. Monitoring of Anti-SARS-CoV-2 Antibodies in People with Undetectable Antibody Levels in the Dot Blot Analysis

To confirm the absence of anti-S-specific antibodies in patient sera that were non-reactive in the dot blot assay against the S1, RBD, and S2 proteins (in total 15/63 patients; Table 1, Supplementary Table S1), the following tests with commercially available immunoassays were performed: Wantai ELISA tests to detect IgM or total antibodies specific for RBD, and the Roche Elecsys anti-S to monitor total anti-S Ig. As controls, sera with restricted anti-S reactivity that was positive for anti-RBD and anti-S2 (patient #46), and anti-S2 (patient #43 and #45) were included in addition to sera with broad anti-S reactivity (positive for anti-S1, anti-RBD, and anti-S2, patients #4, #44, and #47). The control sera were positive using the Wantai anti-RBD and Roche Elecsys anti-S (RBD) immunoassays. Five out of fifteen patients with no detectable anti-S-specific antibodies in the dot blot assay were positive using the Roche Elecsys anti-S (RBD) test (#2, #25, #28, #33, and #34) (Table 1), and twelve out of fifteen patients were positive using the Wantai anti-RBD Ig ELISA (Table 1), highlighting differences in sensitivity between commercially available assays. Importantly, three participants (#11, #42, and #63) with non-reactive sera in the dot blot assay remained negative in the anti-RBD (Wantai) and anti-S Elecsys (Roche) assays at different time points post-symptom onset. It is important to note that patients #11 and #42 had no reported immunosuppressive clinical picture, and patient #63 received Rituximab (Supplementary Table S2). These results indicate that different tests are necessary for accurately determining the anti-SARS-CoV-2 serology of patients with borderline antibody reactivities. Low or undetectable levels of anti-S antibodies in patients indicate that natural infections may not lead to the development of neutralizing antibody titres.

### 3.3. Testing for Neutralizing Capability of Anti-COVID-19 Sera

**Figure 3:**
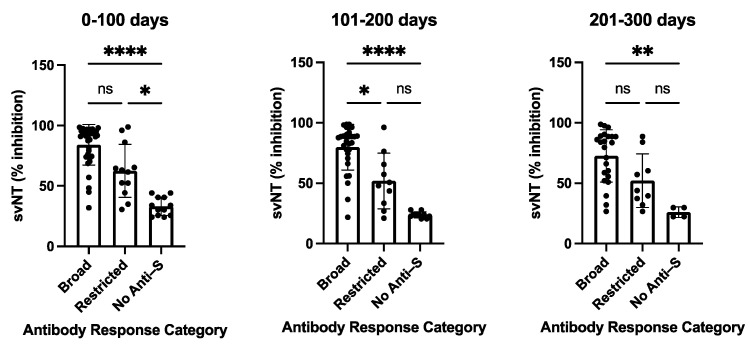
Surrogate virus neutralization test (sVNT) inhibition activities of COVID-19 patients with broad, restricted, and no anti-S antibody levels as measured by dot blot analysis. The sVNT inhibition of sera was taken at the indicated time periods post-symptom onset. The heights of the bar graphs represent the mean values of all individual measurements. Data were analyzed using the analysis of variance (ANOVA) for unpaired samples. Mean values were compared using Dunn’s multiple comparisons test; *p* value: **** < 0.0001; ** < 0.0021; * < 0.0332; not significant (ns) < 0.1234. sVNT: surrogate virus neutralization test (GenScript). Data are also presented in Supplementary Table S3.

The variability of antibody profiles and the absence of detectable levels of anti-S antibodies in participants with a primary SARS-CoV-2 infection led us to assess the neutralizing capability of the antisera. All sera were tested using the in vitro GenScript sVNT assay to measure the inhibition of RBD binding to the ACE2 receptor, followed by testing selected sera with different serological profiles using a cell-culture-based virus neutralization assay. Participants with a broad anti-S antibody profile (*n* = 35/63) developed antibodies that were able to substantially inhibit RBD binding to the ACE2 receptor with a median value of 91.55% (0–100 days post-disease onset, DPSO), 86.05% (101–200 DPSO), and 81.65% (201–300 DPSO). In contrast, lower inhibition efficacies were observed for participants with restricted anti-S responses (*n* = 13) detected in the dot blot assay (57.05%, 45.75%, and 38.4%, for the three time periods 0–100, 101–200, 201–300 DPSO, respectively) or with negative outcomes in the dot blot assay (*n* = 15) (28.2%, 24%, and 11.15%, respectively) (Supplementary Table S3). Sera with a broad anti-S antibody profile in the dot blot assay demonstrated a statistically significant higher neutralizing capability in the sVNT assay than the sera with non-detectable anti-S antibodies in the dot blot assay for all three measured time periods: 0–100, 101–200, and 201–300 days (*p* values vary from <0.0001 to <0.0021) (Figure 3). A trend was observed with a higher neutralizing capability of sera with a broad anti-S antibody profile compared to sera with a restricted anti-S antibody profile (Figure 3).

**Figure 4:**
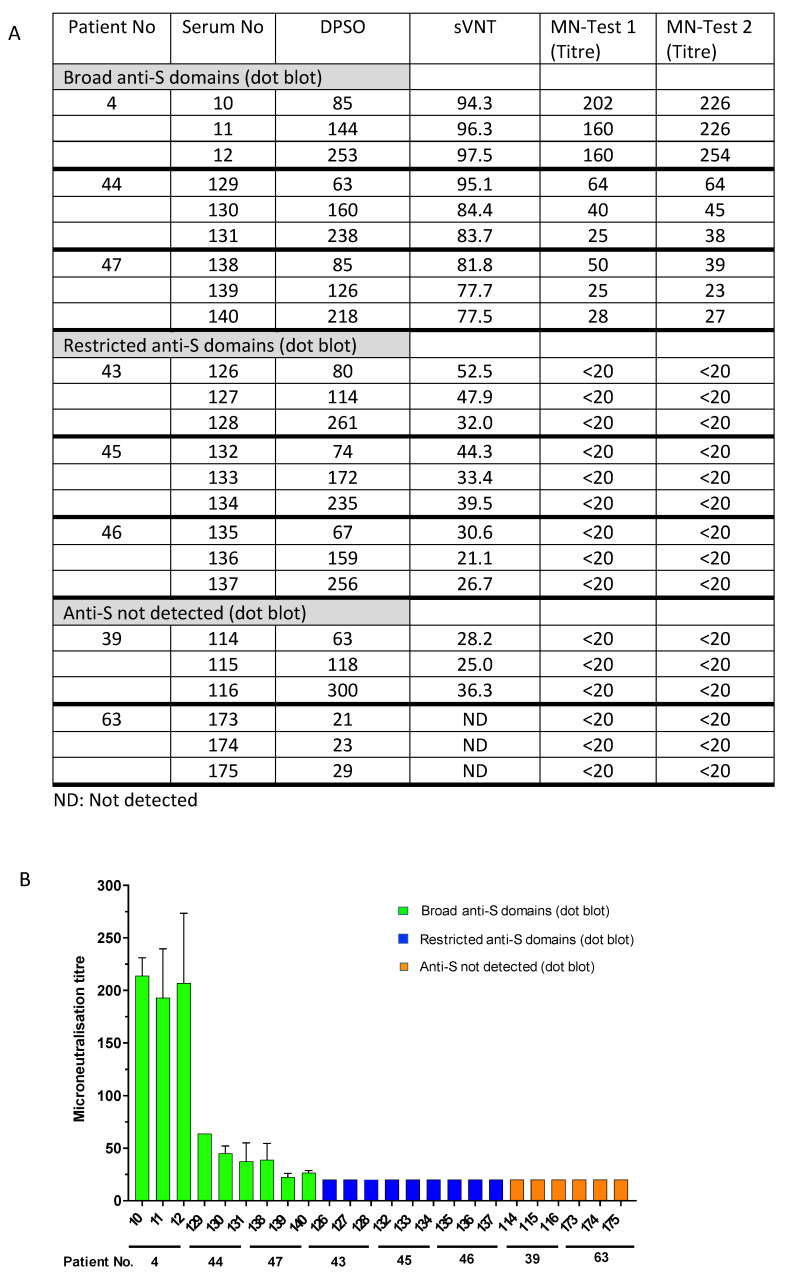
Presence of neutralizing antibodies (microneutralization test, MN) and antibodies inhibiting RBD binding to the ACE2 receptor (surrogate virus neutralization test, sVNT). Comparison of MN titres and binding inhibition (sVNT) in patients with broad anti-S1, -RBA, -S2, and -N antibody responses (patients #4, #47, and #44), narrow antibody responses (patients #43, #45, and #46), and undetected responses (patients #39 and #63). (**A**) The table summarizes the inhibition of RBD binding to ACE2 receptor (sVNT, % inhibition) and titres to neutralize SARS-CoV-2 (MN test). ND: not detected. (**B**). Data are graphed as microneutralization titres. Patient and serum numbers are provided. DPSO: Days after symptom onset indicating when the serum samples were taken (see also legend to Figure 1).

To validate the sVNT results and to determine the reduced virus-neutralizing capability of sera with restricted anti-S antibody responses or undetectable levels of anti-S, selected sera were tested in a cell-culture-based SARS-CoV-2 neutralization assay. The sera #4, #44, and #47, which exhibited broad antibody responses against the different spike protein domains (Figure 1A), inhibited the RBD binding activity to ACE2 in the sVNT assay (ranging from 77.5% to 94.3% (Supplementary Table S3, Figure 3)), and consistently exhibited virus-neutralizing titres from 1:60 to 1:254, 1:25 to 1:64, and 1:23 to 1:50, respectively (Figure 4A). Notably, the neutralizing activity of these sera obtained at different time points after disease onset aligned with the detection of anti-S1/RBD antibodies in the dot blot assay (Figure 1A). However, sera from patients # 43, #45, and #46, which showed a narrow anti-S reactivity in the dot blot assay but detectable levels of anti-RBD antibodies using the Wantai anti-RBD Ig assay (Table 1), showed the inhibition of RBD binding to ACE2 in the sVNT assay (ranging from 21.1% to 52.5%). But, the virus neutralization assay was negative with neutralizing antibody titres below 1:20. Consistently, sera from patients with moderate and severe illness (#39 and #63) in the absence of detectable levels of anti-spike protein antibodies did not show virus neutralization (antibody titres below 1:20, Figure 4). The data indicate that patients do not necessarily develop detectable levels of neutralizing antibodies after infection. Interestingly, the presence of potentially neutralizing antibodies was not associated with the severity of the disease; for instance, participants #44, #43, and #39 suffered moderate COVID-19 but generated variable levels of anti-SARS-CoV-2 neutralizing antibodies. The data strongly suggest that serological follow-up assessments are needed to identify COVID-19 patients who require vaccination after a naturally acquired infection or alternatively to generally provide vaccination after cleared infection to ensure the generation of protective anti-S antibody levels.

## 4. Discussion

Understanding the duration and strength of the anti-SARS-CoV-2 immune response is critical for informing public health strategies and assessing the immune status of the community. Serological surveillance serves as a valuable tool for evaluating the protective immune status of the population, as it can identify individuals with low or non-detectable levels of potentially neutralizing anti-spike protein antibodies, which are crucial for a protective immune status against the virus [39]. We developed a dot-blot assay utilizing different antigenic spike-protein domains (S1, RBD, and S2) to gain insight into the diversity and quality of polyclonal antibody responses against the SARS-CoV-2 spike protein. The analysis of serum samples from a cohort of individuals who were infected with the SARS-CoV-2 Wuhan strain prior to receiving any COVID-19 vaccination revealed patient-to-patient differences in antibody specificities against the antigenic targets S1, RBD, S2, and N proteins. The study outcome indicated that the antibody profiles and antibody signatures observed within the early time period (0–100 days after symptom onset) persisted for up to 300 days, although a reduction in anti-N antibody titres was observed. The microneutralization assay and the in vitro RBD-ACE2 binding inhibition assay (sVNT) showed that patients with a broad anti-spike immune response developed neutralizing antibodies that persisted for more than 300 days after disease onset.

Longitudinal studies have demonstrated varying outcomes from stable antibody titres several months after diagnosis [29,30,32,33,40,41,42,43,44,45] to waning antibody responses [46,47,48,49]. Concerns about immunity post-infection surfaced due to diminished antibody responses. The conflicting outcomes regarding the duration of the humoral immune response are possibly due to variations in the antigen-dependent antibody dynamics and the use of different immunoassays providing different antigenic targets with variable sensitivity thresholds. Also, prolonged duration of virus shedding is associated with long-term antibody positivity in patients and may contribute to differences between different patient cohorts [29,50,51]. In this study, we report that the majority of patients, regardless of disease severity, developed a broad anti-S immune response with antibody specificities for the S1, RBD, and the S2 domain, and importantly, antibodies with the ability to interfere with RBD binding to ACE2 persisted. The data suggest that the development of a broad immune response against the tested S-domains is a good indicator of anti-S antibody persistence, including antibodies with neutralizing activity.

Sera collected between 201 and 300 days post-symptom onset retained their ability to inhibit RBD binding to ACE2 (sVNT), and inhibited virus entry in a microneutralization assay. Similarly, Dispinseri et al. [40] showed that neutralizing antibody titres were detectable for up to eight months in recovered patients, regardless of age or co-morbidities. Related studies confirmed that neutralizing responses can be maintained with a clear correlation between anti-S titres and virus neutralization [42,43,52]. Our data indicated that patients with a broad anti-S antibody response maintain neutralizing activity over a longer time period. This is consistent with the longitudinal profiling of antibody responses against SARS and MERS-CoV, with antibodies detectable more than a year after hospitalization [53,54]. The results also indicated that antibody levels against the individual protein domains (S1, RBD, S2) and the N protein are higher in people who are diagnosed with severe COVID-19 compared to people with mild and moderate COVID-19, which was also observed by Wu et al. [43]. Wu et al. hypothesized that higher antigen levels are present in patients with severe COVID-19, resulting in higher IgG titres. We identified broad anti-S domain antibodies in patients with different disease severities, indicating that the breadth of the antibody response is not correlated with a clinical presentation. Anti-SARS-CoV-2 antibody levels were detected for up to 8 months post-symptom onset, and a broad and sustained polyantigenic immunoreactivity was associated with COVID-19 severity [30,40,41,42,44]. The severity of SARS-CoV-2 infection significantly correlated with higher anti-RBD antibody levels, but suboptimal neutralization potency was a significant predictor of mortality [44]. Sera with a broad anti-S antibody profile in the dot blot assay demonstrated statistically significant higher neutralizing capability in the sVNT assay. We identified broad anti-S domain antibody profiles in patients with different disease severities, indicating that the breadth of the antibody response is not correlated with the clinical presentation but possibly with the neutralizing capacity. Antibody profiling and determination of antibody specificities may contribute to a better understanding of COVID-19-associated clinical outcomes [55]. The decline in anti-N antibody levels is possibly associated with the restricted N antigen availability and accessibility, in contrast to the membrane-associated and exposed S protein. A mild disease progression is possibly associated with reduced virus replication and lower antigen levels, which may facilitate a prominent decline in the patient group with mild disease [43].

In addition to patients with a broad immune response against the spike protein domains and the N protein, we identified patients with low or undetectable antibody levels, as measured by the dot blot assay. Further testing with commercial assays, sVNT, and microneutralization assays confirmed that some patients did not develop detectable levels of antibodies specific for the spike domains and N-protein. Although antibody-mediated protection was compromised in these patients, not all of these patients experienced severe disease, some of them were in the mild categories, and most of them were able to recover, suggesting other mechanisms of counteracting the infection, such as T-cell-mediated immunity or robust innate immunity [56]. Natural infections do not necessarily lead to detectable levels of neutralizing antibodies, highlighting the necessity of establishing the serological status of patients after recovery from COVID-19 [57,58]. The absence of a sample collection at an early time point after symptom onset was a limitation of our study. Lucas et al. [57] demonstrated that the timing of the neutralizing antibody production correlates with the disease trajectory, and COVID-19 mortality correlates with delayed kinetics of neutralizing antibody synthesis. Nevertheless, the presence of anti-S antibodies with neutralizing activities is beneficial.Studies using a neutralizing antibody for the treatment of SARS-CoV-2 infection in preclinical experiments and phase 2 trials resulted in reductions in viral loads in the upper and lower respiratory tracts, fewer hospitalizations, and reduced symptom burden compared to placebo control groups [59].

Our findings illustrate that antibody responses to SARS-CoV-2 exhibit substantial heterogeneity, with notable variations in antibody profiles and polyantigenic immunoreactivity. Notably, naturally infected patients may sustain antibodies that target specific S protein domains and N protein, including neutralizing anti-S antibodies. Neutralizing antibodies could potentially reduce the viral load upon re-infection and contribute to milder disease outcomes. The distinctive signature of the anti-SARS-CoV-2 antibody profile remains stable over time. Patients who exhibit a non-detectable or restricted anti-S antibody response during the initial period after disease onset may potentially benefit from early follow-up immunizations to generate neutralizing antibodies to enhance their ability to neutralize re-infecting SARS-CoV-2.

## Figures and Tables

**Table 1:** Serological tests for anti-SARS-CoV-2 antigens using commercially available assays.

					0–100 Days after Symptom Onset				101–200 Days after Disease Onset				201–300 Days after Disease Onset		
Disease	Patient No.	Age	Dot-Blot	DPSO	Wantai IgM	Wantai RBD	Spike IgG	DPSO	Wantai IgM	Wantai RBD	Spike IgG	DPSO	Wantai IgM	Wantai RBD	Spike IgG
Severity			Outcome		RBD				RBD				RBD		
mild	4	45	S1, RBD, S2	85	D (33.8)	D (21.9)	D (7.0)	144	D (24.6)	D (28.2)	D (6.3)	253	D (16.3)	D (20.9)	D (5.5)
moderate	44	33	S1, RBD, S2	63	D (6.0)	D (21.6)	D (6.7)	160	D (1.5)	D (31.3)	D (4.3)	238	ND	D (21.9)	D (3.2)
moderate	47	33	S1, RBD, S2	85	D (2.5)	D (20.8)	D (4.0)	126	D (1.3)	D (20.8)	D (4.1)	218	ND	D (31.3)	D (3.9)
moderate	46	40	RBD, S2	67	ND	D (7.5)	equiv (1.0)	159	ND	D (12.9)	ND	256	ND	D (13.9)	ND
moderate	43	62	S2	80	ND	D (20.7)	D (2.9)	114	ND	D (20.9)	D (1.7)	128	ND	D (31.3)	equiv (1.0)
moderate	45	76	S2	74	D (1.7	D (16.5)	D (1.7)	172	ND	D (20.5)	equiv (0.9)	235	ND	D (31.3)	D (1.4)
mild	2	37	ND	69	D (10.5)	D (16.8)	D (1.5)	140	D (5.2)	D (19.5)	equiv (0.9)	259	D (8.8)	D (20)	ND
mild	3	51	ND	81	D (1.7)	D (7.6)	equiv (0.9)	147	ND	D (6.3)	ND	247	ND	D (5.9)	ND
mild	11	62	ND	64	ND	ND	ND	118	ND	ND	ND	273	ND	ND	ND
mild	12	43	ND	80	ND	D (10.0)	equiv (1.0)	111	ND	D (14.4)	equiv (1.0)	265	ND	D (21.5)	ND
mild	25	71	ND	74	ND	D (6.0)	D (1.5)	165	ND	D (16.6)	D (1.1)	235	ND	D (21.9)	equiv (1.0)
mild	28	50	ND	59	ND	D (10.0)	D (1.2)					218	ND	D (12.3)	ND
				92	ND	D (9.8)	equiv (1.0)								
mild	32	45	ND	97	ND	D (2.4)	ND	125	ND	D (3.4)	ND				
								188	ND	D (7.0)	ND				
moderate	33	67	ND					117	D (2.1)	D (23.1)	D (4.8)	309	ND	D (22.2)	D (4.4)
moderate	34	64	ND	30	D (1.2)	D (12.6)	D (5.1)	126	ND	D (20.7)	D (2.2)				
moderate	35	40	ND	70	D (4.2)	D (9.5)	ND	115	D (2.4)	D (10.7)	ND	242	D (1.3)	D (16.9)	ND
moderate	39	21	ND	63	ND	D (2.0)	ND	118	ND	D (8.2)	ND	300	ND	D (19.9)	ND
moderate	40	34	ND	65	D (1.3)	D (5.9)	ND	119	equiv (0.9)	D (6.3)	ND	274	ND	D (8.0)	ND
moderate	41	47	ND	60	D (2.4)	D (6.7)	ND	139	D (2.1)	D (19.9)	ND	269	D (2.1)	D (21.1)	ND
moderate	42	35	ND	44	ND	ND	ND	101	ND	ND	ND	236	ND	ND	ND
severe-	63	46	ND	21	ND	ND	ND								
critical				23	ND	ND	ND								
				29	ND	ND	ND								

Sera above the dashed line show anti-SARS-CoV-2 S immunoreactivity in the dot blot assay as indicated in the column “dot-blot outcome”. Sera listed below the dashed line does not show anti-S immunoreactivity in the dot blot assay. The commercially available assays used for re-testing the sera are as follows: Wantai SARS-CoV-2 IgM ELISA, quantitative ELISA for IgG antibody to COVID-19 (Wantai), and Elecsys anti-SARS-CoV-2 S. D: detected; ND: not detected; equiv.: equivocal. DPSO: days post-symptom onset. Numbers in brackets represent signal-to-cut-off ratios.

## Supplementary Materials

The following supporting information can be downloaded at: https://www.mdpi.com/article/10.3390/microorganisms11081985/s1. Table S1: Dot blot assay for the N-protein and the spike protein domains S1, RBD, and S2; Table S2: Participants with no detectable anti-SARS-CoV-2 antibodies in the dot blot analysis; Table S3: Testing of sera with broad anti-S, narrow anti-S or undetectable levels of anti-S antibodies with a surrogate virus neutralization test (sVNT).
