# Supplementary material for: Comparative Longitudinal Serological Study of Anti-SARS-CoV-2 Antibody Profiles in People with COVID-19
Source: Microorganisms. 2023 Aug 2;11(8):1985. doi: 10.3390/microorganisms11081985 (PMC10458948; doi:10.3390/microorganisms11081985)
Supplement: Supplementary file 1 [file microorganisms-11-01985-s001.zip › microorganisms-2448082-supplementary/Suppl Tables S1-S3/Suppl Table S1 enlarged copy.pdf]

Supplementary Table S1. Dot blot assay for the N-protein, and the spike protein domains RBD, S1 and S2.

Disease Severity	Patient No.	Gender	Age	DPSO	0-100 days				Patient No.	DPSO	101-200 days				Patient No.	DPSO	201-300 days			
					Dot Blot Assay (Integrated Density)						Dot Blot Assay (Integrated Density)						Dot Blot Assay (Integrated Density)			
					RBD	S1	S2	N			RBD	S1	S2	N			RBD	S1	S2	N
mild	1	1	43	80	42.6	36.6	104.3	360.8	1	149	129.7	91.8	87.5	257.7	1	262	67.4	44.6	81.0	122.2
mild	2	1	37	69	0.0	0.0	0.0	0.0	2	140	0.0	0.0	0.0	0.0	2	259	0.0	0.0	0.0	0.0
mild	3	1	51	81	0.0	0.0	0.0	0.0	3	147	0.0	0.0	0.0	0.0	3	247	0.0	0.0	0.0	0.0
mild	4	2	45	85	194.1	72.5	262.9	446.1	4	144	229.5	171.6	331.9	169.1	4	253	265.1	172.4	267.6	79.7
mild	5	1	44	19	191.3	0.0	89.0	406.7	5	125	135.7	70.2	0.0	289.9						
mild				34	152.8	70.4	32.6	517.6												
mild	6	1	79	25	107.0	0.0	50.6	677.5												
				41	240.2	15.0	46.8	666.5												
				91	120.4	43.8	162.0	772.3												
mild	7	2	63	38	76.6	91.8	537.0	506.9	7	143	29.9	10.5	308.0	208.4	7	236	0.0	0.0	122.0	114.5
mild	8	2	58	44	62.2	0.0	69.5	147.7							8	213	12.9	0.0	136.1	39.4
				86	55.9	0.0	61.1	141.4												
mild	9	2	49	57	0.0	0.0	64.3	39.3	9	114	0.0	0.0	57.0	32.8	9	273	0.0	0.0	41.1	3.1
mild	10	2	58	66	27.4	0.0	5.9	0.0	10	130	22.8	0.0	0.0	0.0	10	270	16.8	0.0	0.0	0.0
mild	11	2	62	64	0.0	0.0	0.0	0.0	11	118	0.0	0.0	0.0	0.0	11	273	0.0	0.0	0.0	0.0
mild	12	2	43	80	0.0	0.0	0.0	0.0	12	111	0.0	0.0	0.0	0.0	12	265	0.0	0.0	0.0	0.0
mild	13	2	73	81	26.0	0.0	92.8	282.1	13	117	12.3	0.0	73.0	166.4	13	259	19.2	0.0	115.9	9.3
mild	14	2	27	83	57.7	14.5	132.4	583.6	14	124	74.7	75.0	287.1	502.4	14	271	51.2	54.0	183.5	154.3
mild	15	2	39	78	37.3	53.9	128.8	60.9	15	119	21.1	14.7	124.5	39.8	15	303	12.6	9.1	38.0	4.0
mild	16	2	73	50	53.0	20.6	85.4	307.6	16	125	91.4	81.2	98.6	172.2	16	260	66.3	31.0	71.6	52.0
mild	17	2	53	59	0.0	20.0	0.0	127.2	17	150	0.0	4.7	0.0	54.2	17	240	0.0	0.0	0.0	84.6
mild	18	1	51	61	114.6	44.8	283.8	441.2	18	110	86.0	49.6	228.5	330.7	18	229	79.9	54.6	254.9	205.3
mild	19	2	57	69	229.8	78.3	259.1	559.0	19	144	262.6	130.8	135.2	380.6	19	237	166.2	121.6	131.9	234.1
mild	20	2	21	71	109.5	56.0	60.3	212.0	20	169	86.2	40.3	46.7	186.5	20	231	86.1	40.2	39.2	90.9
mild	21	2	82	69	85.5	48.6	267.4	578.5	21	144	95.9	49.7	234.6	472.9	21	237	128.3	83.2	355.1	466.0
mild	22	1	27	61	21.9	26.9	145.8	52.3	22	152	42.1	31.3	128.3	44.3	22	257	67.6	54.3	158.1	58.3
mild	23	1	77	56	101.7	62.7	95.9	694.0	23	154	73.9	24.5	57.5	628.2	23	217	37.6	14.4	24.8	387.8
mild	24	1	79	73	0.0	0.0	262.1	413.7	24	117	0.0	0.0	215.4	199.5						
									24	178	0.0	0.0	181.8	75.1						
mild	25	1	71	74	0.0	0.0	0.0	109.2	25	165	0.0	0.0	0.0	42.7	25	235	0.0	0.0	0.0	68.0
mild	26	1	78	73	40.2	90.3	197.5	458.9	26	138	238.5	169.2	138.4	327.6	26	247	254.7	59.3	96.2	181.0
mild	27	2	20	45	73.5	6.2	23.6	327.8	27	117	67.2	21.0	103.3	206.5	27	226	108.7	40.6	192.9	155.7
mild	28	1	50	59	0.0	0.0	0.0	0.0							28	218	0.0	0.0	0.0	0.0
				92	0.0	0.0	0.0	0.0												
mild	29	2	58	48	227.3	109.1	212.3	446.1	29	111	215.6	98.4	195.7	169.5	29	202	117.4	87.8	179.9	88.9
mild	30	1	82	80	74.2	35.4	125.8	404.4	30	106	38.3	30.3	106.7	351.0						
									30	155	107.0	59.0	164.8	343.8						
									30	185	44.6	29.8	62.7	185.7						
mild	31	2	31	83	0.0	0.0	121.4	0.0	31	124	0.0	8.7	132.1	0.0	31	216	0.0	0.0	94.0	0.0
mild	32	2	45	97	0.0	0.0	0.0	0.0	32	125	0.0	0.0	0.0	0.0						
										188	0.0	0.0	0.0	0.0						
moderate	33	2	67						33	117	0.0	0.0	0.0	0.0	33	309	0.0	0.0	0.0	0.0
moderate	34	1	64	30	0.0	0.0	0.0	0.0	34	126	0.0	0.0	0.0	0.0						
moderate	35	1	40	70	0.0	0.0	0.0	0.0	35	115	0.0	0.0	0.0	0.0	35	242	0.0	0.0	0.0	0.0
moderate	36	1	67	43	81.9	15.5	114.5	354.7	36	129	25.8	0.0	29.4	162.8	36	231	72.6	0.0	65.2	134.4
moderate	37	2	67	42	58.6	32.1	76.1	390.2	37	134	120.2	48.4	86.4	496.3	37	238	121.0	27.3	60.8	266.4
moderate	38	1	59	56	0.0	0.0	67.4	236.1	38	133	0.0	0.0	32.0	108.5	38	253	0.0	0.0	43.9	101.9
moderate	39	2	21	63	0.0	0.0	0.0	0.0	39	118	0.0	0.0	0.0	0.0	39	300	0.0	0.0	0.0	0.0
moderate	40	2	34	65	0.0	0.0	0.0	0.0	40	119	0.0	0.0	0.0	0.0	40	274	0.0	0.0	0.0	0.0
moderate	41	2	47	60	0.0	0.0	0.0	0.0	41	139	0.0	0.0	0.0	0.0	41	269	0.0	0.0	0.0	0.0
moderate	42	1	35	44	0.0	0.0	0.0	0.0	42	101	0.0	0.0	0.0	0.0	42	236	0.0	0.0	0.0	0.0
moderate	43	1	62	80	0.0	0.0	15.8	710.9	43	114	19.2	0.0	28.4	687.9	43	261	19.2	0.0	5.9	416.1
moderate	44	2	33	63	80.2	9.2	536.5	646.2	44	160	40.5	13.6	338.5	255.0	44	238	67.2	23.4	290.8	74.4
moderate	45	2	76	74	0.0	0.0	141.9	102.2	45	172	0.0	0.0	195.7	31.6	45	235	0.0	0.0	279.7	19.2
moderate	46	1	40	67	79.4	0.0	147.0	379.7	46	159	49.0	0.0	125.9	331.6	46	256	52.9	0.0	113.4	207.1
moderate	47	1	33	85	200.9	89.4	268.5	739.4	47	126	163.1	88.2	331.7	845.4	47	218	126.1	83.0	371.6	462.6
moderate	48	2	35	61	280.3	142.3	249.6	721.9	48	152	381.4	229.5	135.4	702.3						
				89	408.7	188.2	363.3	712.4	48	188	384.2	199.7	346.7	675.7						
moderate	49	2	76	83	101.6	61.1	107.9	630.5	49	111	47.7	20.8	11.0	533.2						
										174	97.9	20.1	0.0	366.2						
severe	50	1	61						50	152	45.5	89.6	234.9	598.1	50	228	141.7	87.0	252.5	527.2
severe	51	1	75						51	139	109.6	74.6	179.2	735.5	51	247	78.9	74.4	163.3	414.1
severe	52	1	87						52	151	263.5	98.7	265.4	457.1	52	243	258.6	98.1	216.0	281.8
severe	53	1	71						53	145	95.0	87.7	117.6	732.5	53	233	53.5	70.8	130.2	676.6
severe	54	1	42	42	362.3	98.0	246.9	761.7							54	252	291.4	95.8	324.6	285.9
severe	55	2	36	39	24.4	0.0	80.8	139.6							55	215	27.3	88.7	195.6	0.0
severe	56	2	84	87	302.4	91.8	240.7	614.7	56	156	184.0	88.5	251.0	415.4						
severe-critical	57	2	56						57	161	113.2	89.1	197.8	66.9	57	245	90.2	88.4	131.1	170.7
severe-critical	58	1	72	49	60.0	97.1	173.9	221.9	58	110	279.0	98.7	241.5	417.2						
severe-critical	59	2	61	93	332.9	96.8	91.9	613.8	59	170	189.3	88.5	78.5	446.3						
severe-critical	60	1	55	90	315.7	95.6	428.9	508.5												
severe-critical	61	1	72	16	76.7	90.8	187.5	828.5												
				22	118.4	93.5	169.8	744.1												
severe-critical	62	2	57	22	32.3	94.5	467.0	540.3												
				27	6.5	95.3	450.5	614.8												
severe-critical	63	1	46	21	0.0	0.0	0.0	0.0												
				23	0.0	0.0	0.0	0.0												
				29	0.0	0.0	0.0	0.0												

Patients showing immunoreactivity against the four antigens, N, S1, RBD and S2 are highlighted in **blue**; sera with no reactivity against the four antigens are highlighted in **green**, sera with resticted antibody profiles in **black**.

DPSO: Days Post-Symptoms Onset
