# Supplementary material for: Comparative Longitudinal Serological Study of Anti-SARS-CoV-2 Antibody Profiles in People with COVID-19
Source: Microorganisms. 2023 Aug 2;11(8):1985. doi: 10.3390/microorganisms11081985 (PMC10458948; doi:10.3390/microorganisms11081985)
Supplement: Supplementary file 1 [file microorganisms-11-01985-s001.zip › microorganisms-2448082-supplementary/Suppl Tables S1-S3/Suppl Table S2.pdf]

Supplement Table S2

Participants with no detectable anti-COVID-19 antibodies in the dot blot analysis

| Participant | Co-morbidities |
|---|---|
| **02** | No documented co-morbidities |
| **03** | No documented co-morbidities |
| **11** | Smoker, bladder infection, Keflex (Cephalosporin antibiotic), Hiprex PRN |
| **12** | No documented co-morbidities |
| **28** | Gout, obesity, hypertension |
| **32** | Thyroid Hashimoto’s disease, Eutroxsig |
| **33** | Benign pituitary tumor, resection 2015, Thyroxine |
| **34** | No documented co-morbidities |
| **35** | Smoker |
| **39** | No documented co-morbidities |
| **40** | Smoker |
| **41** | No documented co-morbidities |
| **42** | No documented co-morbidities |
| **63** | Chronic kidney disease, renal tumor, follicular non-Hodgkin lymphoma on remission, **Rituximab**, Moxifloxacin, Clexane, Remdesivir |

PRN: pro re nata
