# Supplementary material for: Comparative Longitudinal Serological Study of Anti-SARS-CoV-2 Antibody Profiles in People with COVID-19
Source: Microorganisms. 2023 Aug 2;11(8):1985. doi: 10.3390/microorganisms11081985 (PMC10458948; doi:10.3390/microorganisms11081985)
Supplement: Supplementary file 1 [file microorganisms-11-01985-s001.zip › microorganisms-2448082-supplementary/Suppl Tables S1-S3/Suppl Table S3.pdf]

Suppl. Table S3. Testing of sera with broad-anti-S, narrow anti-S or undetectable levels of anti-S antibodies with an receptor binding inhibition assay (sVNT)

Broad anti-S	Patient No.	Age	DPSO	0-100 days<br>sVNT	DPSO	101-200 days<br>sVNT	DPSO	201-300 days<br>sVNT
mild	**1**	43	80	**91.8**	149	**83.4**	262	**79.7**
mild	**4**	45	85	**94.3**	144	**96.3**	253	**97.5**
mild	**5**	44	19	**92.3**	125	**81.0**		
			34	**91.3**				
mild	**6**	79	25	**68.7**				
			41	**70.3**				
			91	**79.3**				
mild	**7**	63	38	**91.1**	143	**82.8**	236	**83.6**
mild	**14**	27	83	**74.1**	124	**70.5**	271	**71.2**
mild	**15**	39	78	**ND**	119	**ND**	303	**ND**
mild	**16**	73	50	**31.9**	125	**21.8**	260	**ND**
mild	**18**	51	61	**88.3**	110	**92.1**	229	**86.1**
mild	**19**	57	69	**98.6**	144	**98.2**	237	**96.3**
mild	**20**	21	71	**73.1**	169	**66.2**	231	**58.7**
mild	**21**	82	69	**93.1**	144	**90.3**	237	**84.7**
mild	**22**	27	61	**44.9**	152	**56.0**	257	**55.5**
mild	**23**	77	56	**76.3**	154	**55.8**	217	**50.9**
mild	**26**	78	73	**96.8**	138	**96.4**	247	**90.4**
mild	**27**	20	45	**57.1**	117	**50.0**	226	**52.0**
mild	**29**	58	48	**87.5**	111	**77.9**	202	**66.0**
mild	**30**	82	80	**48.3**	106	**36.6**		
					155	**35.6**		
					185	**24.6**		
moderate	**37**	67	42	**97.4**	134	**96.5**	238	**97.4**
moderate	**44**	33	63	**95.1**	160	**84.4**	238	**83.7**
moderate	**47**	33	85	**81.8**	126	**77.7**	218	**77.5**
moderate	**48**	35	61	**95.7**	152	**87.7**		
			89	**95.3**	188	**88.5**		
moderate	**49**	76	83	**74.5**	111	**89.1**		
					174	**98.7**		
severe	**50**	61			152	**88.5**	228	**87.0**
severe	**51**	75			139	**74.6**	247	**74.4**
severe	**52**	87			151	**98.7**	243	**98.1**
severe	**53**	71			145	**87.7**	233	**70.8**
severe	**54**	42	42	**98.0**			252	**95.8**
severe	**56**	84	87	**91.8**	156	**88.5**		
severe-critical	**57**	56			161	**89.1**	245	**88.4**
severe-critical	**58**	72	49	**97.1**	110	**98.7**		
severe-critical	**59**	61	93	**96.8**	170	**88.5**		
severe-critical	**60**	55	90	**95.6**				
severe-critical	**61**	72	16	**90.8**				
			22	**93.5**				
severe-critical	**62**	57	22	**94.5**				
			27	**95.3**				
				Median 91.55<br>IQR (95.3-74.2)		***Median 86.05<br>IQR (90.75-63.65)***		***Median 81.65<br>IQR (89.90-60.53)***
**Restricted-anti-S**								
mild	**8**	58	44	**65.3**			213	**37.3**
			86	**52.4**				
mild	**9**	49	57	**ND**	114	**ND**	273	**ND**
mild	**10**	58	66	**ND**	130	**ND**	270	**ND**
mild	**13**	73	81	**61.6**	117	**50.7**	259	**43.9**
mild	**17**	53	59	**63.2**	150	**65.6**	240	**57.2**
mild	**24**	79	73	**64.5**	117	**57.3**		
					178	**43.6**		
mild	**31**	31	83	**34.9**	124	**26.9**	216	**ND**
moderate	**36**	67	43	**98.8**	129	**96.2**	231	**83.9**
moderate	**38**	59	56	**86.2**	133	**76.3**	253	**60.3**
moderate	**43**	62	80	**52.5**	114	**47.9**	261	**32.0**
moderate	**45**	76	74	**44.3**	172	**33.4**	235	**39.5**
moderate	**46**	40	67	**30.6**	159	**21.1**	256	**26.7**
severe	**55**	36	39	**96.10**			215	**88.7**
				Median 57.05<br>IQR (70.53-33.83)		***Median 45.75<br>IQR (63.53-22.55)***		***Median 38.40<br>IQR (59.53-6.68)***
**Anti-S Not Detected**								
mild	**2**	37	69	**40.5**	140	**27.9**	259	**22.3**
mild	**3**	51	81	**44.2**	147	**24.1**	247	**ND**
mild	**11**	62	64	**ND**	118	**ND**	273	**ND**
mild	**12**	43	80	**25.7**	111	**23.9**	265	**29.8**
mild	**25**	71	74	**30.3**	165	**20.6**	235	**ND**
mild	**28**	50	59	**33.9**			218	**ND**
			92	**24.2**				
mild	**32**	45	97	**ND**	125	**22.7**		
					188	**27.2**		
moderate	**33**	67			117	**82.6**	309	**95.2**
moderate	**34**	64	30	**76.9**	126	**ND**		
moderate	**35**	40	70	**36.6**	115	**37.1**	242	**33.3**
moderate	**39**	21	63	**28.2**	118	**25**	300	**36.3**
moderate	**40**	34	65	**21.8**	119	**20**	274	**ND**
moderate	**41**	47	60	**44.3**	139	**50.4**	269	**47**
moderate	**42**	35	44	**ND**	101	**ND**	236	**ND**
severe-critical	**63**	46	21	**ND**				
			23	**ND**				
			29	**ND**				
				Median 28.2<br>IQR (40.5-28.2)		***Median 24<br>IQR (30.2-15)***		***Median 11.15<br>IQR (35.55-0)***

ND: Not detected; calculated as inhibition 0%
